# Numerical Simulation of Temperature Field Distribution During Directional Annealing of TiAl Alloy with Different Parameters

**DOI:** 10.3390/ma18071537

**Published:** 2025-03-28

**Authors:** Feng Huang, Yeyu Hu, Jiaguo Xu, Zhili Hu, Yanxiong Liu, Lin Hua

**Affiliations:** 1 Hubei Key Laboratory of Advanced Technology of Automotive Components, Wuhan University of Technology, Wuhan 430070, China; huangfeng@whut.edu.cn (F.H.); yyhooray571428@163.com (Y.H.); 17855265299@163.com (J.X.); hualin@whut.edu.cn (L.H.); 2 Hubei Longzhong Laboratory, Xiangyang 441022, China

**Keywords:** directional annealing, TiAl alloy, numerical simulation, temperature field

## Abstract

In this paper, a bidirectional temperature gradient directional annealing process for growing TiAl columnar crystals was proposed, and the influences of structural parameters and process parameters on the temperature distributions of TiAl rods were discussed through numerical simulation. The results indicate that the α phase zone is expanded and its boundary becomes planar as the thickness of graphite ring (b) and gap width (d) decrease. Increasing the graphite rod length (l) and the height of the graphite ring from the Ga-In coolant surface (h) results in an expanded α phase zone with flattened boundaries, but the temperature gradient decreases. Taking all the α phase zone height, its boundary shape, and the temperature gradient into consideration, the optimal b, d, l, and h are 10 mm, 5 mm, 50 mm, and 50 mm, respectively. The higher heating temperature within the α phase temperature range, such as 1375 °C, is favorable for the establishment of the required temperature field during directional annealing. The effect of drawing speed is more complicated. Although its effect on the temperature field of the TiAl rod is almost negligible, it will seriously affect the microstructure of the annealed alloy, and it needs to be optimized by subsequent experiments.

## 1. Introduction

Ti-based and Al-based alloys have broad prospects in the aerospace and automotive industries [1]. In particular, due to the advantages of low density (3.9~4.2 g/cm^3^, depending on composition), high specific strength, high specific stiffness, temperature resistance, corrosion resistance, oxidation resistance [2,3,4], etc., TiAl alloy is considered to be an excellent material for manufacturing aero engine blades used in the range of 650~1000 °C [5] so as to improve the thrust–weight ratio of aero engine and Mach number of new-generation aircraft. Since 4822 (Ti-48Al-2Cr-2Nb) was successfully applied to the last two stages of the low-pressure turbine blades of the Boeing 787 GEnx-1B engine [6], the research and application of other TiAl alloys, such as TNM (Ti-43.5Al-4Nb-1Mo-0.1B), TNB (Ti-45Al-5/8Nb-0.2B-0.2C), 45XD (Ti-45Al-2Mn-2Nb-0.8 vol%TiB_2_) and high Nb-TiAl alloy, are also accelerating [7]. In the TiAl alloy casting process, intrinsic defects such as shrinkage cavities, microporosity, elemental segregation, and microcracks are inevitably formed within the microstructure. Conventional heat treatment methods have been demonstrated to show limited effectiveness in modifying the as-cast structure. Although partial defect elimination can be achieved through thermal processing, significant enhancement of high-temperature mechanical properties remains unattainable. This limitation has been attributed to the persistence of equiaxed grain morphology throughout the treatment process. Compared with equiaxed crystal, directional cylindrical crystal can greatly improve the service performance of TiAl alloy blades because the transverse grain boundary sensitive to holes and cracks is eliminated and the stress axis is parallel to the grain boundary [8].

Directional solidification (DS) is widely utilized for growing directional columnar and single crystals. In TiAl alloy processing, multiple established techniques have been employed, including the Bridgman method [9], optical floating zone melting (FZM), electromagnetic constraining shaping (EMCS) [10], and cold crucible directional solidification (CCDS) [11]. The process typically consists of three sequential phases: thermal treatment, melting, and controlled solidification. However, The engineering implementation of directionally solidified TiAl alloys has remained constrained by the inherent challenges associated with the elevated chemical reactivity of their molten states [12]. These limitations are primarily manifested in three aspects: (1) intricate process design requirements, (2) stringent restrictions on specimen dimensions/geometries, and (3) operational complexities in achieving precise solidification control [13,14]. Specifically, the Bridgman method often results in coating erosion and alloy contamination [15,16]. FZM and EMCS greatly limit the sample size, which is hardly larger than 15 mm [17,18]. In CCDS, the stable growth of columnar grains is easily disturbed by electromagnetic stirring [19].

Differing from directional solidification, directional annealing can realize regional and dynamic heat treatment. By controlling the migration behaviors of grain and phase boundaries during directional annealing, it is possible to obtain the required directional columnar crystals by directional grain growth and directional solid phase transformation in a pure solid state [20]. Obviously, because the long-term directional growth process is carried out in a pure solid state, it is not affected by the high activity of TiAl alloy melt, and the size and shape of the sample treated are unlimited. Based on this, directional annealing is a promising technology for growing directional columnar or even single crystals of TiAl alloy. Chen et al. [21,22,23,24] explored the microstructure evolution and mechanical properties of TiAl alloy during directional annealing under electromagnetic induction zone heating, and achieved directional grain growth and mechanical properties improvement. However, the columnar crystals grown at present are still small in length to diameter ratio and discontinuous. Meanwhile, in the previous experiment, the axial forced cooling was only applied to the lower part of the sample, while the upper part had free heat dissipation.

The establishment of controlled directional heat dissipation conditions, fundamentally governed by the axial temperature gradient magnitude, serves as the critical determinant for the growth of columnar crystals in metallic systems [25]. In the process of directional annealing, if the axial temperature gradient can be established in both the heating process of the sample entering the heating zone and the cooling process of the sample leaving the heating zone, it is more conducive to the acquisition of columnar crystals. Based on this, a bidirectional temperature gradient directional annealing model was established in this paper, and the influences of the structure parameters of the directional annealing device and directional annealing process parameters on the temperature field of the TiAl sample were discussed to provide a theoretical basis and data support for the subsequent experiments.

## 2. Bidirectional Temperature Gradient Directional Annealing Principle

**Figure 1:**
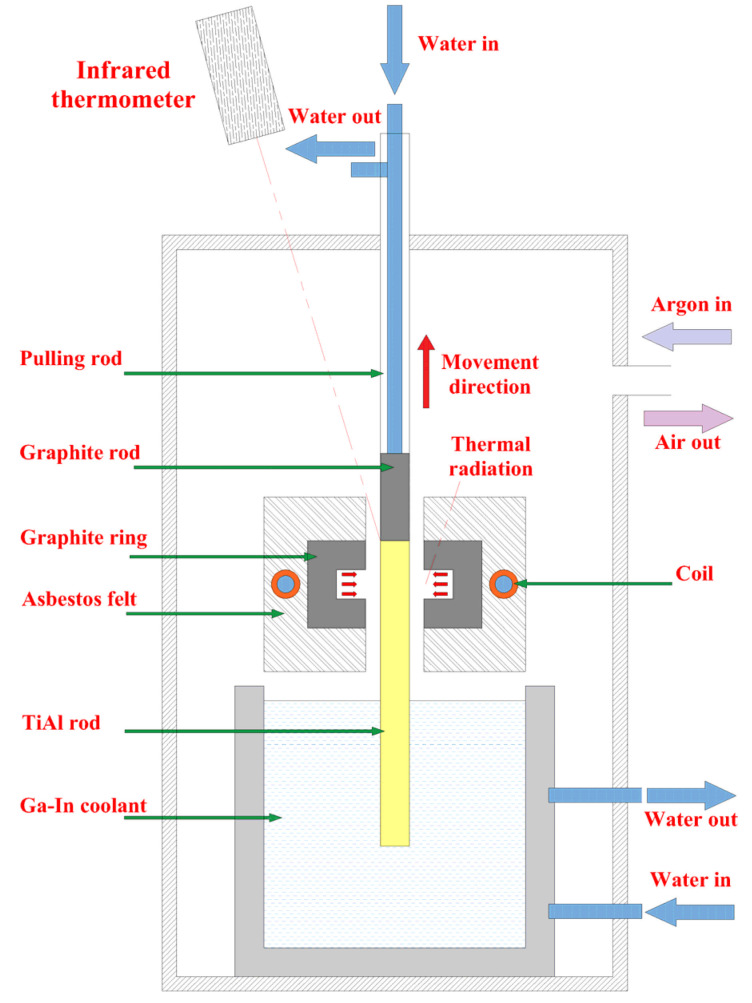
Schematic diagram of bidirectional temperature gradient directional annealing.

Figure 1 shows the schematic diagram of bidirectional temperature gradient directional annealing. As illustrated, the graphite ring is heated by electromagnetic induction, and then the sample is heated regionally by the thermal radiation of the graphite ring. Asbestos felt outside the graphite ring is used to provide insulation and reduce lateral heat dissipation. In the axial direction, the lower part of the sample is cooled by Ga-In coolant under the heating zone to establish the lower axial temperature gradient. Meanwhile, the upper part of the sample is cooled by circulating water in the pulling rod to established the upper axial temperature gradient. The sample moves upward with the pulling rod, and it goes through the process of entering the heating zone for heating and then leaving the heating zone for cooling. Under the action of the upper and lower axial temperature gradient, the directional grain growth and directional solid phase transformation are carried out, and then columnar crystals are obtained. In order to avoid the oxidation of TiAl alloy during directional annealing, the whole process is carried out under the protection of argon gas.

## 3. Numerical Model of Directional Annealing

### 3.1. Establishment of Model

**Figure 2:**
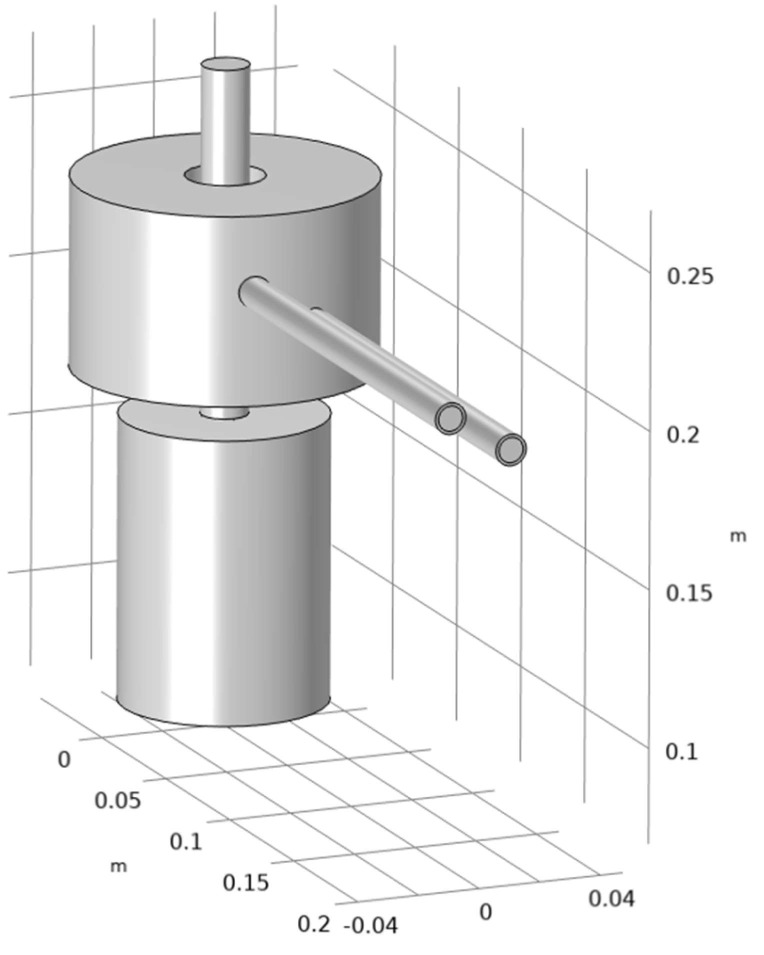
Three-dimensional simulation model for TiAl alloy rod bidirectional temperature gradient directional annealing.

Based on the principle mentioned above, a finite element numerical model for bidirectional temperature gradient directional annealing was established, as shown in Figure 2. The upper end of the treated TiAl rod is connected to the water-cooled pulling rod through a graphite rod, and the lower end is immersed in a liquid Ga-In alloy. Considering the existing conditions in the laboratory and the subsequent directional annealing experiment plan, the diameter and length of the TiAl alloy rod in this finite element model are set to 15 mm and 100 mm, respectively. For induction heating, skin effects should be considered in the meshing process [26]. Based on this, a boundary layer mesh was set on the surface of the TiAl rod.

In addition, in order to avoid excessive or even difficult computations due to the excessive complexity of the model, several assumptions were made to simplify the model while maintaining accuracy: (1) Material properties are assumed to be uniform and isotropic. (2) The initial environmental temperature within the simulation was set at 298.15 K. (3) The boundaries of the system are assumed to be thermally insulated. (4) Material deformation during heating is neglected. (5) Suppose that the thermal radiation between the graphite ring and the TiAl alloy rod in the directional annealing furnace is the gray body thermal radiation satisfying the Boltzmann equation.

### 3.2. Simulation Parameters

After constructing the geometric model, the material properties of each domain within the model need to be defined. The reasonable setting of physical property parameters is a key factor affecting the accuracy of the numerical simulation results. The material property parameters used in this paper are mainly derived from the literature [27,28,29], as shown in Table 1.

### 3.3. Governing Equations and Boundary Conditions

As shown in Figure 1, the directional annealing process involves induction heating of the graphite ring using a high-frequency (30 kHz) electromagnetic field generated by an induction coil, which in turn radiates heat to the treated TiAl alloy rod. Meanwhile, the sample is cooled by both the Ga-In coolant at the bottom and the graphite rod at the top. Based on this, using the Comsol Multiphysics 6.2 software, a finite element model composed of a magnetic field, solid and fluid heat transfer, surface-to-surface radiation, and turbulence was established.

Under the action of the thermal effect of the induced eddy currents, the temperature of the graphite ring increases rapidly. The joule heat generated by induced eddy currents on the graphite ring is used as the internal heat source and transferred to the interior through thermal radiation and thermal conduction. According to Joule’s law [30], in the unit volume of the graphite ring, the joule heat generated by the induced eddy currents per unit time can be calculated by the following formula:

(1) $$q=\frac{Je^{2}}{\sigma }$$

where *q* is the internal heat source strength, measured in W/m^3^; *σ* is the conductivity of the graphite ring, measured in S/m; and *Je* is the induced vortex density, measured in A/m^2^.

The Fourier thermal conduction differential equation of the induction heating process without convective heat transfer is as follows [31,32].

(2) $$\frac{\partial T}{\partial t}=\frac{\lambda }{\rho C_{p}}\left(\frac{\partial^{2}T}{\partial x^{2}+\frac{\partial^{2}T}{\partial y^{2}}}\right)+\frac{q}{\rho C_{p}}$$

where *T* is the temperature, *t* is the time, *λ* is the thermal conductivity, *q* is the internal heat source strength, and *ρ* is the density of TiAl alloy. The time derivative term is ignored in the steady-state simulation.

Unlike heat convection and conduction, radiative heat transfer does not require a medium. Heat radiation is the process by which objects exchange energy in space in the form of electromagnetic waves. In this process, the electromagnetic energy emitted by one object is absorbed by other objects and converted into heat energy, so radiative heat transfer does not require any medium. According to Stefan–Boltzmann law [33], the heat radiation could be typically expressed as follows:

(3) $$j=\varepsilon \sigma_{b}T^{4}$$

where *j* is the energy radiated per unit surface area of the radiator in unit time, measured in W/m^2^; *ε* is the emissivity, representing the ability of the radiator surface to radiate heat, with a value between 0 and 1; and *σ_b_* is the Stefan–Boltzmann constant, which is about 5.67 × 10^−8^, measure in W/(m^2^·k^4^).

Before the start of the calculation, the boundary conditions applied according to the cooling system and laboratory environment are as follows: the initial temperature of the top surface of the graphite rod, the TiAl rod, the circulating cooling water, and the Ga-In coolant were set to 303.15 K, while the ambient temperature was set to 298.15 K. As shown in Figure 1, since the lower part of the TiAl rod is immersed in the Ga-In coolant, the heat exchange between them can be represented as [34,35]

(4) $$P=\delta (T-T_{Ga-In})$$

where *P* is the heat flow density, measured in J; *δ* is the coefficient of heat conduction, measured in W/m^2^K; and *T* is the temperature of TiAl rod and *T_Ga-In_* is the temperature of Ga-In coolant, both measured in K.

The inductive coil is cooled by circulating cooling water. Based on this, the coil’s heat dissipation can be simulated by using an effective high thermal conductivity coefficient, combined with a uniform extraneous convective loss term, as shown in the following formula [36].

(5) $$Q=\frac{\frac{dM}{dt}C_{p}(T_{in}-T)}{2\pi rA}$$

where *dM*/*dt* is the mass flow rate of the cooling water, measured in kg/s; *C_p_* is the specific heat capacity of water, measured in J/(kg·K); *T_in_* is the inlet temperature of the water and *T* is the temperature of the induction coil, both measured in K; *r* represents the inner radius of the induction coil, measured in m; and *A* is the cross-sectional area of the cooling channel, measured in m^2^.

## 4. Results and Discussion

**Figure 3:**
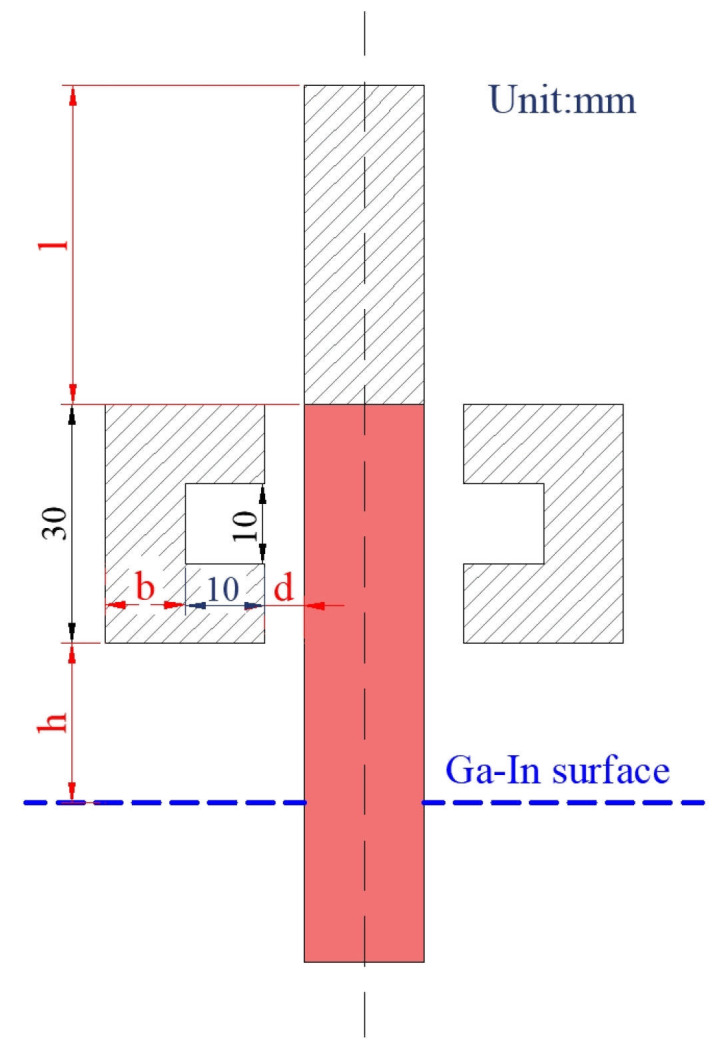
Structural parameters investigated.

In order to provide guidance for subsequent bidirectional temperature gradient annealing experiments, the effects of structural parameters and process parameters on temperature distribution were simulated and analyzed. As shown in Figure 3, the structural parameters include the thickness of the graphite ring (b), the length of the graphite rod (l), the height at which the bottom of the graphite ring leaves from the Ga-In coolant surface (h), and the width of the gap between the TiAl rod and the graphite ring (d). As illustrated in Table 2, the control variable method was used to analyze the effects of the above four structural parameters on the temperature field during this directional annealing process. Considering the size of the existing electromagnetic induction melting equipment and the size of the prepared TiAl alloy ingot (height: 80~120 mm; diameter: 15~30 mm), reasonable parameters were designed for each structure parameter. For the process parameters, the heating zone temperature (hereinafter referred to as heating temperature) and drawing speed were mainly investigated.

### 4.1. Effects of Structural Parameters

**Figure 4:**
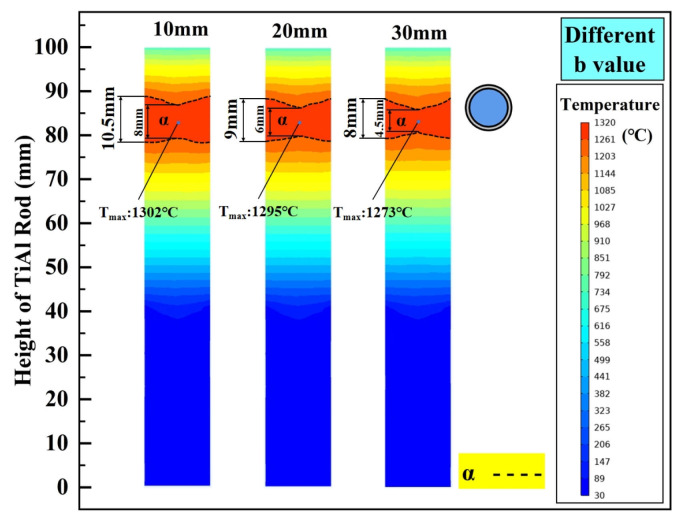
The influence of b on the steady-state temperature field of the TiAl rod.

According to reference [37], the temperature range of α single phase of Ti-43.5Al (at%) alloy is 1260~1380 °C. Meanwhile, since TNM (Ti-43.5Al-4Nb-1Mo-0.1B) will be used as the experimental object in the subsequent experimental study, 1320 °C was selected as the target heating temperature when analyzing the effects of structural parameters. The heating zone is defined as the localized high-temperature region on the TiAl bar, generated through direct thermal radiation from the graphite ring. This zone maintains a stable temperature profile aligned with the α single phase, providing critical thermodynamic driving forces for complete γ → α phase transformation. In the actual simulation, this heating temperature was obtained by applying a power load of 6.2 kW to the induction coil. Figure 4 shows the temperature distribution characteristics under different b, where the black dashed line represents the isotherm where the temperature of the TiAl alloy rod reaches Tα. As illustrated, the thickness of the graphite ring has a great influence on the temperature field of the TiAl alloy during directional annealing. When b is 10 mm, the position where the TiAl rod aligns with the central height of the graphite ring forms an α phase temperature region with a maximum height of 10.5 mm. The boundaries of this zone exhibited relatively planar characteristics, with a height difference of 2.5 mm between the widest and narrowest sections. In contrast, when b is increased to 20 mm, a 14.3% reduction in the α phase zone height (9 mm) is observed, accompanied by more pronounced boundary fluctuations (height difference: 3 mm). Further increasing b to 30 mm results in a continued decline of the α phase zone height to 8 mm (23.8% reduction compared to b = 10 mm), along with even more evident undulations (height difference: 3.5 mm). Obviously, Under the same heating power, the inner wall temperature of the graphite ring decreases with increasing b, reducing the radiation intensity to the TiAl rod. Thus, the temperature at the center of the heating zone, T_max_, decreases significantly with increasing b. Under the above conditions, the optimal graphite ring thickness for achieving a stable α phase temperature zone with minimal boundary fluctuations is identified as 10 mm. Of course, at higher heating power, a thicker graphite ring can theoretically achieve this effect, but this will inevitably lead to an increase in energy consumption.

**Figure 5:**
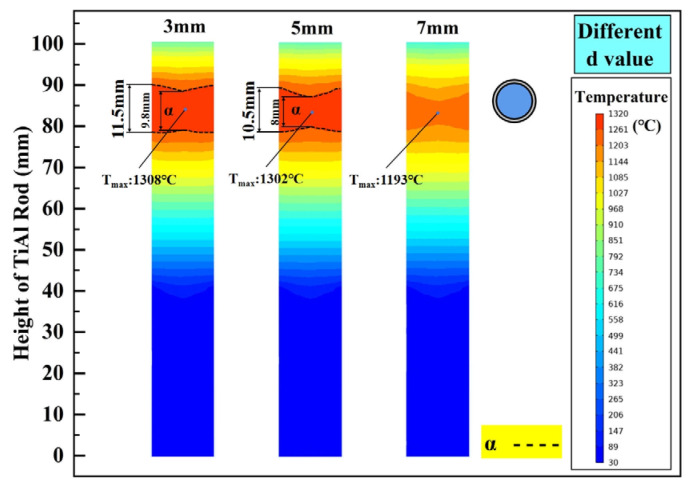
The influence of d on the steady-state temperature field of the TiAl rod.

In addition to b, the gap width d between the graphite ring and TiAl rod will also affect the thermal radiation intensity acting on the treated sample. Setting the gap width to be 3 mm, 5 mm, and 7 mm, its influence on the internal temperature distribution of the TiAl rod is investigated, and the calculated results are shown in Figure 5. As illustrated, with the increase in gap width, the height of the α phase zone decreases and the boundary fluctuation becomes more pronounced. When the gap width increases to 7 mm, under this heating power, the heating zone of the TiAl rod does not reach the α phase zone temperature, and its maximum temperature is only 1193 °C (8.4% reduction compared to d = 3 mm). Obviously, for obtaining a steady α phase and flat isothermal interface, the smaller the gap width, the better. However, as shown in Figure 1, too narrow a gap will result in difficulty in measuring the hot zone temperature with an infrared thermometer. Based on this, 5 mm is considered to be the most appropriate gap width.

**Figure 6:**
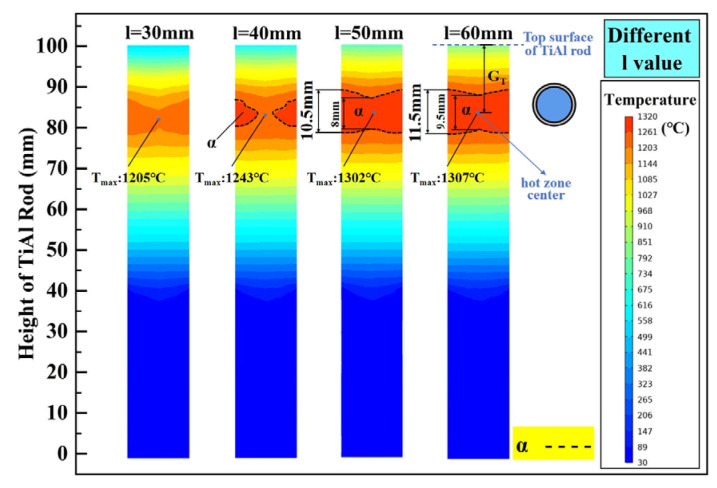
The influence of l on the steady-state temperature field of the TiAl rod.

The temperature gradient (G_T_) is an important factor affecting the directional grain growth and directional solid transformation during directional annealing [38]. As illustrated in Figure 3, the graphite rod length (l) and the height of the graphite ring from the Ga-In coolant surface (h) are the key factors affecting the upper and lower temperature gradient, respectively. By setting l to be 30 mm, 40 mm, 50 mm, and 60 mm, its influence on the internal temperature distribution of the TiAl rod is investigated, and the calculated results are shown in Figure 6. When l is 30 mm, resulting from the strong cooling from the circulating water in the pulling rod, the heating zone of the TiAl rod does not reach the α phase zone temperature under this heating power (6.2 kW), and its maximum temperature is only 1205 °C. Obviously, the cooling effect of the circulating cooling water on the TiAl rod decreases with the increase in l. As l is increased to 40 mm, although the α phase zone appears in the heating zone of the TiAl rod, it is not complete in cross-section. When l reaches 50 mm, a complete α phase zone is formed on the cross-section of the heating zone of the TiAl rod, and the further increase in l results in an expansion of the α phase zone and a flatter α phase boundary. It should be noted that the increase in l will inevitably lead to a decrease in the temperature gradient at the upper part of the TiAl rod. Taking the average temperature gradient between the hot-zone center and top surface of the TiAl rod into consideration, its value under different l conditions is 37.83 °C/mm, 35.12 °C/mm, 32.86 °C/mm, and 31.13 °C/mm, respectively. Taking all the α phase zone height, its boundary shape, and the temperature gradient into consideration, the optimal graphite rod length (l) is believed to be 50 mm.

**Figure 7:**
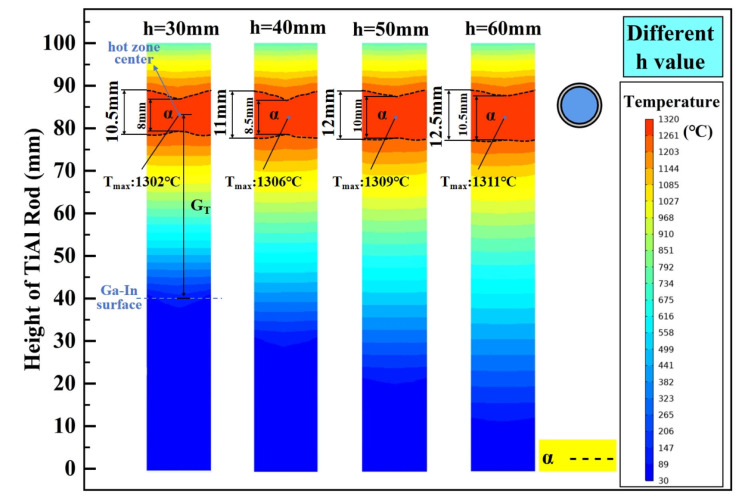
The influence of h on the steady-state temperature field of the TiAl rod.

The temperature gradient at the lower end of the TiAl rod can be adjusted by varying the height of the graphite ring from the Ga-In coolant surface (h). Setting h to be 30 mm, 40 mm, 50 mm, and 60 mm, its influence on the internal temperature distribution of the TiAl rod is investigated, and the calculated results are shown in Figure 7. As illustrated, because the chilling effect of the Ga-In coolant on the TiAl rod is weakened, and the heat absorbed by the coolant from the TiAl rod is reduced, the height of the α phase zone in the TiAl rod increases with the increase in h, and its boundary becomes flatter. It should be noted that the temperature gradient at the front of the heating zone is crucial for directional annealing. A higher temperature gradient can inhibit the recrystallization of grains before they enter the heating zone, thereby accumulating more energy at grain boundaries and preserving the driving force for columnar grain growth [39]. Taking the average temperature gradient between the hot-zone center and Ga-In coolant surface into consideration, its value under different h conditions is 25.73 °C/mm, 21.96 °C/mm, 18.62 °C/mm, and 15.69 °C/mm, respectively. Taking all the α phase zone height, its boundary shape, and the temperature gradient into consideration, the optimal height of the graphite ring from the Ga-In coolant surface (h) is believed to be 50 mm.

### 4.2. Effects of Process Parameters

**Figure 8:**
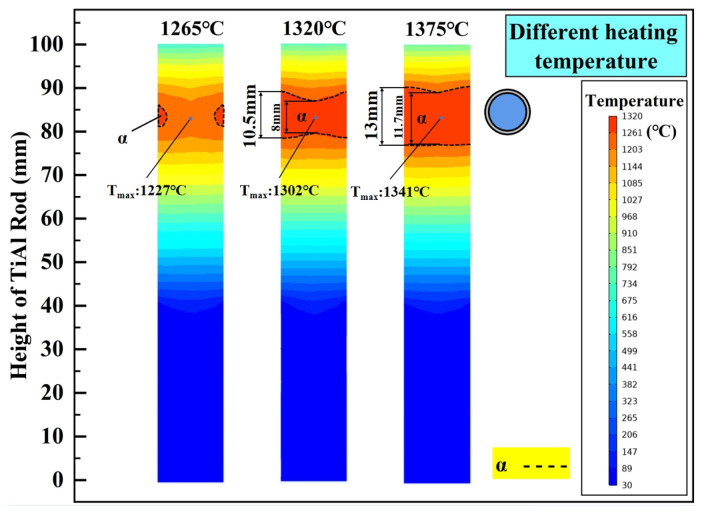
Effect of heating temperature on steady-state temperature field of TiAl rod.

Heating temperature and drawing speed are two key process parameters affecting directional annealing. In order to explore the temperature distribution characteristics of the TiAl rod during directional annealing at different hot zone temperatures, 1265 °C, 1320 °C, and 1375 °C were selected as the steady-state temperatures in the core hot zone for simulation. The calculated results are shown in Figure 8. As illustrated, when the heating temperature is 1265 °C, attributing to the insufficient thermal radiation intensity, only a small part of the outer surface of the TiAl rod exceeds the α phase transformation temperature. Obviously, this is not allowed. In theory, achieving a uniform α single phase over the entire cross-section of the TiAl rod is critical for governing grain growth during directional annealing. When the heating temperature is elevated to 1320 °C, a complete α phase zone spanning the entire cross-section is achieved, reaching a maximum height of 10.5 mm. However, significant boundary fluctuations can be observed, and the height difference in the α phase zone boundaries is recorded as 2.5 mm. Further increasing the heating temperature to 1375 °C results in expanding the α phase zone and smoothing the corresponding boundaries. Compared with that under 1320 °C, the maximum height increases by 23.8% to 13 mm, while the height difference decreases by 48% to 1.3 mm. A planar α phase interface facilitates competitive grain growth and suppresses grains with non-preferred crystallographic orientations [40]. Based on this, higher heating temperatures within the Tα range of the TiAl alloy strongly favor the formation and growth of columnar grains. Compared with the temperature field of directional annealing with unidirectional temperature gradient [28], the boundary of the core hot zone in this paper is flatter (heating temperature = 1375 °C), and the boundaries of upper and lower temperature zones are flatter. Furthermore, according to Arrhenius’ relationship [39], the heating temperature affects the migration rate of grain boundaries. The higher the heating temperature, the faster the grain boundary migration [41,42].

**Figure 9:**
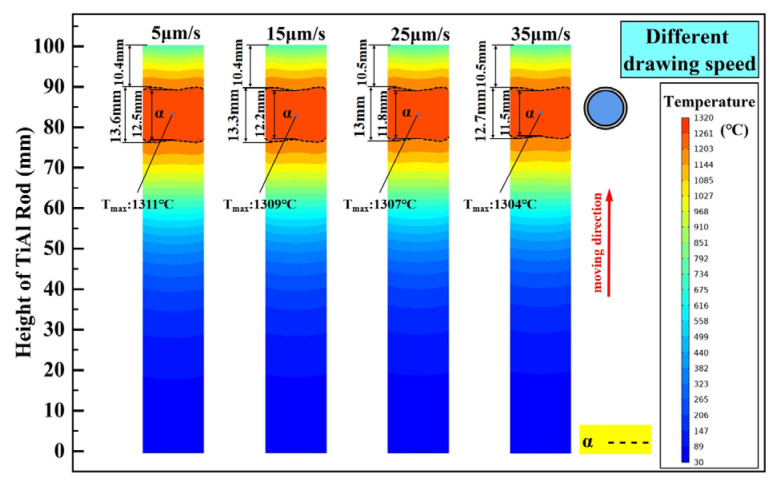
Effect of drawing speed on steady-state temperature field of TiAl rod.

As shown in Figure 1, the TiAl rod is pulled upwards at a certain speed and passes successively through the hot zone to complete directional annealing. Obviously, the drawing speed will also affect the temperature field of the TiAl rod. Based on the above static discussions and setting the drawing speed to be 5 μm/s, 15 μm/s, 25 μm/s, and 35 μm/s, its influence on the temperature field of TiAl rod during directional annealing is investigated, and the calculated results are shown in Figure 9. As illustrated, the maximum height of the α phase zone decreases slightly with increasing drawing speed, which decreases from 13.6 mm to 12.7 mm as the drawing speed increases from 5 μm/s to 35 μm/s. Notably, under different drawing speeds, the height difference in the α phase zone boundaries exhibits negligible variation, with a fluctuation of only 0.1 mm. Obviously, in the range of parameters selected above, the influence of drawing speed on the temperature field of the TiAl rod during directional annealing can be ignored.

It should be pointed out that the drawing speed is closely related to the grain growth behaviors of the TiAl alloy [43]. Holm [44] proposed that to explain the influence of drawing speed on columnar grain growth during directional annealing, the relationship between drawing speed and grain boundary migration rate must be considered. Experimental evidence supporting Holm’s theory was provided by Zhang [45] through their investigation of microstructural evolution in industrial pure iron during directional annealing

When the drawing speed is extremely low, the grain boundary migration rate exceeds the drawing speed. In this case, grains grow normally through conventional coarsening, with minimal directional annealing effects on grain boundary migration. In the extreme scenario of a zero drawing speed (0 μm/s), directional annealing degenerates into isothermal annealing within the thermal zone, resulting in the formation of coarse equiaxed grain structures. Therefore, when the drawing speed remains below the grain boundary migration rate, the aspect ratio of the grains increases with the rising drawing speed [46]. When the drawing speed is excessively high (far exceeding the grain boundary migration rate), the thermal zone rapidly traverses the specimen, leaving insufficient time to influence crystallization orientation. Consequently, grains still develop into equiaxed structures through normal growth. However, the accelerated drawing speed reduces the dwell time in the thermal zone, leading to limited grain growth. Therefore, when the drawing speed remains above the grain boundary migration rate, the aspect ratio of grain decreases with the increase in the drawing speed [47,48]. Columnar grain structures emerge only when the drawing speed approximates the grain boundary migration rate. Under this condition, grain boundaries can migrate synchronously with the thermal zone. Moreover, the aspect ratio of columnar grains increases with closer matching between the drawing speed and grain boundary migration rate. Theoretically, when the growth interface of the columnar crystal moves synchronously with the hot zone, that is, when the growth rate of the columnar crystal is equal to the drawing speed, the aspect ratio of the grains can be maximized.

Therefore, while the drawing speed exhibits negligible influence on the temperature field of the TiAl alloy during directional annealing, it critically governs the microstructural evolution (e.g., grain aspect ratio and crystallographic texture). Consequently, the precise calibration of this parameter necessitates subsequent experimental validation combined with post-annealing microstructural characterization.

## 5. Conclusions

In this paper, a bidirectional temperature gradient directional annealing model was established, the temperature distribution characteristics of the TiAl rod were simulated, and the influences of structural and process parameters were discussed. The main conclusions can be summarized as follows:

The structural parameters b, d, l, and h of the bidirectional temperature gradient directional annealing device have significant effects on the temperature field of the TiAl bar during annealing. To achieve a flat α phase boundary and maximize the temperature gradient on the TiAl rod during directional annealing, providing sufficient driving force for initial columnar grain growth, the corresponding optimized parameters are believed to be 10 mm, 5 mm, 50 mm, and 50 mm, respectively.

The maximum height of the α phase zone increases with increasing heating temperature, while its boundary fluctuation decreases. Higher heating temperatures within the Tα range of TiAl alloy, such as 1375 °C, are favorable for the formation and growth of columnar grains.

Although drawing speed has little effect on the temperature field of the TiAl rod during directional annealing (evidenced by minimal variations in α phase zone height is only about 0.1 mm), it will significantly affect the microstructure of the annealed alloy. Previous studies demonstrated that columnar grain formation and aspect ratio maximization occur when the drawing speed matches the grain boundary migration rate, enabling synchronized boundary thermal zone movement. These findings highlight the necessity of optimizing drawing speed to balance thermal stability and microstructural control, necessitating subsequent experiments to validate the optimized parameters.

Model simplification was implemented through selected assumptions, which may introduce computational deviations in simulation accuracy. Material isotropy and homogeneity will be systematically analyzed using crystallographic characterization methods. The influence of graphite ring thermal deformation on directional annealing temperature fields will be quantified through coupled thermodynamic simulations.

## Figures and Tables

**Table 1:** Material property parameters.

Material Parameter	Copper	Graphite	TiAl Alloy	Asbestos	Ga-In	Units
Relative permeability	1	1	1	1	1	-
Thermal conductivity	400	105 × (T/300)^−0.3^ × exp(−3.5 × 10^−4^ (T − 300))	21.7959 + 8.1633 × 10^−3^T	0.12	16.5	W/(m·K)
Density	8960	1950	3850	1000	6350	kg/m^3^
Heat capacity at constant pressure (Cp)	385	710	1040	800	306.6	J/(kg·K)
Electrical resistivity	1.67 × 10^−8^	6.29 × 10^−6^	6.6588 × 10^−7^ + 0.79 × 10^−9^T − 8.16 × 10^−13^T^2^ + 5.72 × 10^−12^T^3^	-	9.39 × 10^−6^	Ω·m

**Table 2:** Structural parameters used in the controlled variable method experiments (unit: mm).

Parameters	b	h	l	d
Effect of b	10, 20, 30	30	50	5
Effect of d	10	30	50	3, 5, 7
Effect of l	10	30	30, 40, 50, 60	5
Effect of h	10	30, 40, 50, 60	50	5

## Data Availability

The original contributions presented in this study are included in the article. Further inquiries can be directed to the corresponding authors.
